# Effects of Artificial Intelligence-Derived Body Composition on Kidney Graft and Patient Survival in the Eurotransplant Senior Program

**DOI:** 10.3390/biomedicines10030554

**Published:** 2022-02-26

**Authors:** Nick Lasse Beetz, Dominik Geisel, Seyd Shnayien, Timo Alexander Auer, Brigitta Globke, Robert Öllinger, Tobias Daniel Trippel, Thomas Schachtner, Uli Fehrenbach

**Affiliations:** 1 Department of Radiology, Charité—Universitätsmedizin Berlin, Corporate Member of Freie Universität Berlin and Humboldt-Universität zu Berlin, 13353 Berlin, Germany; dominik.geisel@charite.de (D.G.); seyd.shnayien@charite.de (S.S.); timo-alexander.auer@charite.de (T.A.A.); uli.fehrenbach@charite.de (U.F.); 2 DZHK (German Center for Cardiovascular Research), 10785 Berlin, Germany; tobias_daniel.trippel@charite.de; 3 Berlin Institute of Health, 10178 Berlin, Germany; brigitta.globke@charite.de; 4 Department of Surgery, Charité—Universitätsmedizin Berlin, Corporate Member of Freie Universität Berlin and Humboldt-Universität zu Berlin, 13353 Berlin, Germany; robert.oellinger@charite.de; 5 Department of Internal Medicine—Cardiology, Charité—Universitätsmedizin Berlin, Corporate Member of Freie Universität Berlin and Humboldt-Universität zu Berlin, 10117 Berlin, Germany; 6 Division of Nephrology, University Hospital Zurich, 8091 Zürich, Switzerland; thomas.schachtner@usz.ch

**Keywords:** kidney transplant, transplantation, Eurotransplant Senior Program (ESP), body composition, computed tomography (CT), artificial intelligence (AI)

## Abstract

The Eurotransplant Senior Program allocates kidneys to elderly transplant patients. The aim of this retrospective study is to investigate the use of computed tomography (CT) body composition using artificial intelligence (AI)-based tissue segmentation to predict patient and kidney transplant survival. Body composition at the third lumbar vertebra level was analyzed in 42 kidney transplant recipients. Cox regression analysis of 1-year, 3-year and 5-year patient survival, 1-year, 3-year and 5-year censored kidney transplant survival, and 1-year, 3-year and 5-year uncensored kidney transplant survival was performed. First, the body mass index (BMI), psoas muscle index (PMI), skeletal muscle index (SMI), visceral adipose tissue (VAT), and subcutaneous adipose tissue (SAT) served as independent variates. Second, the cut-off values for sarcopenia and obesity served as independent variates. The 1-year uncensored and censored kidney transplant survival was influenced by reduced PMI (*p* = 0.02 and *p* = 0.03, respectively) and reduced SMI (*p* = 0.01 and *p* = 0.03, respectively); 3-year uncensored kidney transplant survival was influenced by increased VAT (*p* = 0.04); and 3-year censored kidney transplant survival was influenced by reduced SMI (*p* = 0.05). Additionally, sarcopenia influenced 1-year uncensored kidney transplant survival (*p* = 0.05), whereas obesity influenced 3-year and 5-year uncensored kidney transplant survival. In summary, AI-based body composition analysis may aid in predicting short- and long-term kidney transplant survival.

## 1. Introduction

In an aging society, frailty is one of the largest challenges facing healthcare as patients who suffer from sarcopenia, cachexia, and obesity are at risk for prolonged hospitalization, perioperative complications, and poorer overall survival [1,2]. Therefore, appropriate identification of these patients at risk is desirable. Unlike the body mass index (BMI), artificial intelligence (AI)-based analysis of body composition can differentiate the relative proportions of various tissues using muscle and adipose tissue parameters including the skeletal muscle index (SMI), psoas muscle index (PMI), visceral adipose tissue (VAT), and subcutaneous adipose tissue (SAT) [3]. Additionally, body composition analysis can be used to detect sarcopenia, which is defined as the presence of low muscle mass using sex specific cut-off values, and sarcopenic obesity, which is defined as the combined presence of both sarcopenia and obesity [4]. The metabolic information derived from this kind of individual body composition analysis can identify frail patients (e.g., patients with sarcopenic obesity who have a normal BMI with reduced muscle mass and severe obesity) [5].

Kidney transplant recipients aged 65 years and older with end-stage renal disease benefit from being allocated a transplant kidney in the Eurotransplant Senior Program (ESP) by having a better survival rate and quality of life compared with that from hemodialysis treatment [6,7]. Despite the survival benefit, kidney transplant recipients still have a high mortality rate compared with that of the general population [8]. Organ shortage and perioperative morbidity necessitate the careful workup of potential kidney transplant recipients to ensure an optimal outcome. Aside from cardiovascular disease, arterial hypertension, and diabetes mellitus, preoperative frailty has been shown to be associated with a higher risk of death and delayed graft function [9,10]. Additionally, surgical complications and delayed graft function are more common in obese patients [11,12].

Especially in older and diabetic patients, a computed tomography (CT) scan of the abdomen and pelvis is routinely performed to detect iliac calcification as peripheral vascular disease increases the risk of transplant ischemia [13]. In general, the information gained from the CT is only used to plan optimal graft positioning [14]. However, these imaging studies could easily be used for additional preoperative analysis of body composition as well [15]. CT body composition parameters have been identified as outcome predictors in many cardiovascular and oncological diseases. For example, body composition has been reported to predict life-threatening progression of aortic enlargement in Marfan syndrome or severe complications, prolonged hospitalization, and overall survival in esophageal cancer [16,17]. Moreover, abdominal obesity has been found to predict coronary heart disease, and the PMI seems to predict outcomes in patients undergoing transcatheter aortic valve implantation [18,19].

The hypothesis of this study is that AI-based body composition parameters may influence 1-year, 3-year and 5-year patient survival, 1-year, 3-year and 5-year censored kidney transplant survival, and 1-year, 3-year and 5-year uncensored kidney transplant survival in elderly kidney transplant recipients from the ESP. As initial evaluation of older candidates for kidney transplant routinely includes CT examinations of the abdomen and pelvis, we used the CT data for a retrospective AI-based analysis of individual body compositions to identify possible imaging predictors and imaging risk factors for patient and transplant survival.

## 2. Materials and Methods

### 2.1. Study Design

In this single-center study, we analyzed body composition in a retrospective dataset of kidney transplant recipients from the ESP who had undergone a CT scan of the abdomen and pelvis for initial evaluation before transplantation. The study was approved by the institutional review board and performed in compliance with the Declaration of Helsinki.

### 2.2. Patient Population and Characteristics

Kidney transplant recipients from the ESP aged 65 years and older were included in this study if they had undergone CT of the lower abdomen and pelvis for initial evaluation. They were referred for CT from the Department of Surgery or the Department of Nephrology. All patients included underwent solitary kidney transplantation at our university transplant center between 2011 and 2016. Exclusion criteria were patients without CT scans prior to surgery and aged <65 years. Additionally, patients were excluded if their CT images did not include the third lumbar vertebra.

### 2.3. Baseline Data

Two patients had to be excluded because the CT scan of their lower abdomen and pelvis did not include the third lumbar vertebra. A total of 42 patients with a mean age of 69 ± 4 years at the time of transplantation (range: 65 to 80 years) were included in this study: 13 women and 29 men. The mean weight was 78 ± 14 kg, and mean height was 171 ± 8 cm. BMI was calculated using the following formula: BMI = weight/height^2^ (kg/m^2^): mean BMI was 27 ± 7.

Almost all patients received basiliximab for induction and Tac/MMF/MP for maintenance of immunosuppression. One patient received rATG for induction and another patient received CyA/everolimus/MP for permanent immunosuppression. Further clinical characteristics of kidney transplant recipients and donor organs are compiled in Table 1.

### 2.4. Data Collection, Follow-Up, and Endpoints

All data were retrieved from the patient records and clinical database. Thirty-one kidney transplant recipients attended aftercare at our university transplant center at 3-month intervals, and 11 kidney transplant recipients were followed up by local nephrologists or general practitioners. Follow-up rates at 1 year, 3 years, and 5 years after transplantation were 100%. Endpoints were defined as 1-year, 3-year, and 5-year patient survival and 1-year, 3-year, and 5-year censored transplant survival and uncensored (= not censored for death) transplant survival.

### 2.5. Body Composition Analysis

**Figure 1:**
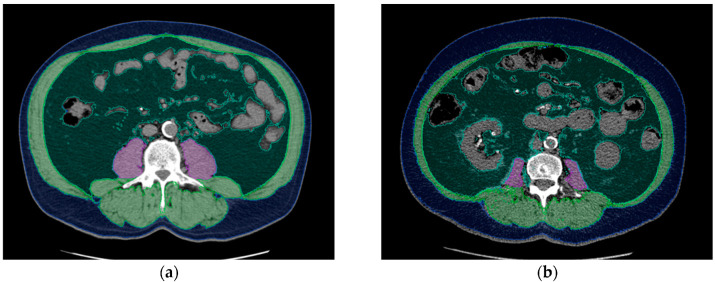
Examples of AI-based analysis of body composition: (**a**) 65-year-old male kidney transplant recipient with a BMI of 27.7, PMI of 9.8, and SMI of 67.6. (**b**) 65-year-old male kidney transplant recipient with a BMI of 27.8, PMI of 3.9, and SMI of 44.4. Even though both patients are the same age and have almost the same BMI, their body composition parameters are considerably different. Each segmented tissue is coded with a different color: psoas muscle = purple, skeletal muscle (except psoas muscle) = green, visceral fat = dark green, blue = subcutaneous fat. Tissue density and area were automatically calculated using Visage version 7.1.

Available CT datasets acquired at the Department of Radiology and at external locations were used for analysis of body composition. Image segmentation was performed with an AI-based automated software tool using a convolutional neural network, U-net (Visage version 7.1, Visage Imaging GmbH, Berlin, Germany). The network consists of nine blocks: four down-sampling blocks, four up-sampling blocks, and one in between. The training data consisted of 200 axial CT images of the L3 level, and augmentation was applied during training to improve generalization of the network. Psoas muscle, skeletal muscle, visceral adipose tissue, and subcutaneous adipose tissue were automatically separated and coded with different colors. Automatic segmentation was checked by an experienced radiologist. In few cases, AI-based image segmentation was manually corrected, for example, when hypodense stool in the intestine was misinterpreted as body fat. The software automatically calculated the area in square centimeters (cm^2^) and density in Hounsfield units of each segmented tissue class. Areas of skeletal muscle, visceral adipose tissue (VAT), and subcutaneous adipose tissue (SAT) at L3 were derived for body composition analysis. The psoas muscle index (PMI) was calculated using the following formula: psoas muscle area (cm^2^)/body surface area (m^2^). The skeletal muscle index (SMI) was calculated using the following formula: skeletal muscle area (cm^2^)/body surface area (m^2^). Examples of AI-based automated analysis of body composition are shown in Figure 1.

### 2.6. Induction and Maintenance Immunosuppression

The choice of induction therapy was based on immunologic risk. Kidney transplant recipients with a low immunologic risk received an interleukin-2 receptor blockade with basiliximab, and recipients with a high immunologic risk received lymphocyte-depleting induction with rATG. Primary immunosuppression consisted of a triple-drug combination of a calcineurin inhibitor (CNI), tacrolimus or cyclosporine, antimetabolite (mycophenolate mofetil (MMF), mycophenolic acid (MPA) or azathioprine), and steroids. Steroid treatment was tapered over 8 weeks to a dose of 4 mg methylprednisolone/day.

### 2.7. Statistical Analysis

For analysis of 1-year, 3-year, and 5-year patient survival and kidney transplant survival, multivariate Cox regression was performed, and *p* ≤ 0.05 was considered to indicate a significant difference. Kaplan–Meier curves were plotted for 3-year and 5-year uncensored kidney transplant survival and log-rank testing was performed. All data analyses were performed using IBM SPSS Statistics version 27 (International Business Machines Corporation, IBM, Armonk, NY, USA).

For each outcome endpoint, including 1-year, 3-year, and 5-year patient survival and 1-year, 3-year, and 5-year censored transplant survival and 1-year, 3-year, and 5-year uncensored transplant survival, the dependent variant was defined as death of the patient or kidney transplant. First, BMI and the AI-derived body composition parameters PMI, SMI, VAT, and SAT served as independent variants. Second, cut-offs for sarcopenia, defined as SMI ≤ 38.5 cm^2^/m^2^ in women and SMI ≤ 52.4 cm^2^/m^2^ in men, and obesity (BMI ≥ 30), were used as independent variates. Age as a possible confounder was excluded. Sex was included by defining specific cut-offs for men and women [20].

## 3. Results

### 3.1. AI-Based Body Composition Parameters and Cut-Offs

All AI-based body composition parameters were derived at the third lumbar vertebra level. The mean PMI was 5.4 ± 1.9 cm^2^/m^2^, and the mean SMI was 42.0 ± 7.6 cm^2^/m^2^. The mean VAT was 203.0 ± 123.3 mm^2^, and SAT 204.7 ± 91.2 mm^2^. Thirteen patients (31%) had sarcopenia, 11 patients (26%) had obesity, and 5 patients (12%) had sarcopenic obesity. All results are compiled in Table 2.

### 3.2. Cox Regression Analysis with BMI and AI-Derived Body Composition Parameters as Independent Variates

One-year, 3-year, and 5-year patient survival was not predicted by BMI or any AI-derived body composition parameter including PMI, SMI, VAT and SAT. All results are compiled in Table 3.

One-year censored kidney transplant survival was significantly predicted by a reduced PMI (*p* = 0.03) and reduced SMI (*p* = 0.03); 3-year censored kidney transplant survival was significantly predicted by a reduced SMI (*p* = 0.05); but 5-year censored kidney transplant survival was not predicted by BMI or any AI-derived body composition parameter. All results are compiled in Table 4.

One-year uncensored kidney transplant survival was significantly predicted by a reduced PMI (*p* = 0.02) and reduced SMI (*p* = 0.01). In contrast, 3-year uncensored kidney transplant survival was significantly predicted by increased VAT (*p* = 0.04), while 5-year uncensored kidney transplant survival was not predicted by BMI or any AI-derived body composition parameter. All results are compiled in Table 5.

### 3.3. Cox Regression Analysis with Sarcopenia and Obesity as Cut-Off Independent Variates

Cut-off values for sarcopenia and obesity did not influence 1-year, 3-year or 5-year patient survival. The results are compiled in Table 6.

Cut-off values for sarcopenia and obesity did not influence 1-year, 3-year or 5-year censored kidney transplant survival. The results are compiled in Table 7.

Cut-off values for sarcopenia significantly predicted uncensored 1-year uncensored kidney transplant survival (*p* = 0.05), and the values for obesity significantly predicted 3-year and 5-year uncensored kidney transplant survival. The results are compiled in Table 8. 

**Figure 2:**
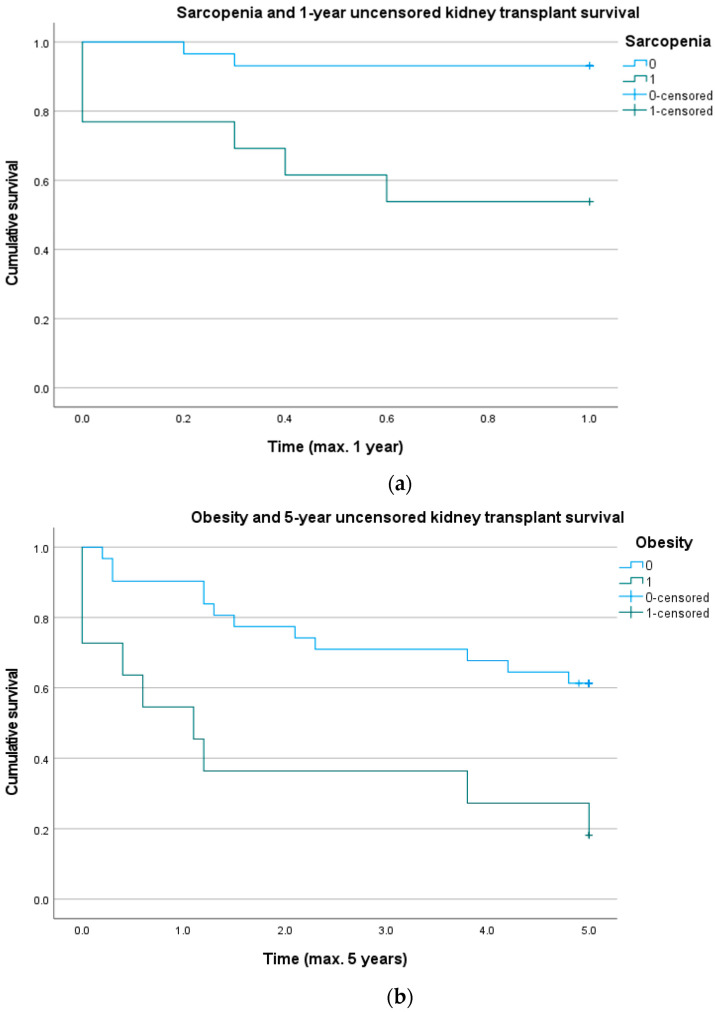
(**a**) Graph showing that sarcopenia assessed by a routine pretransplant computed tomography (CT) scan may influence 1-year uncensored graft survival. Log rank test: *p* = 0.002. (**b**) Graph showing that obesity may influence 5-year uncensored graft survival. Log rank test: *p* = 0.007.

The influence of sarcopenia on 1-year uncensored kidney transplant survival and the influence of obesity on 5-year uncensored kidney transplant survival are shown in Kaplan–Meier curves (Figure 2). The log-rank showed the significant influence of sarcopenia and obesity (*p* = 0.002 and *p* = 0.007, respectively).

## 4. Discussion

In this retrospective study we evaluated the usefulness of artificial intelligence-based body composition analysis for predicting kidney transplant survival and recipient survival in the Eurotransplant Senior Program. Outcome endpoints were defined as 1-year, 3-year, and 5-year for patients and transplants. Body composition parameters were tested as possible outcome predictors by analyzing overall patient and transplant survival over a follow-up period of 5 years after kidney transplantation. For 1-year censored and uncensored transplant survival, we identified lower a PMI and SMI as significant predictors of graft loss. The 3-year uncensored kidney transplant survival was significantly influenced by higher VAT, whereas 3-year censored kidney transplant survival was significantly influenced by a lower SMI. Moreover, sarcopenia significantly predicted 1-year uncensored transplant survival. Long-term 3-year and 5-year survival analysis identified obesity as a significant risk factor for uncensored transplant survival. In contrast, recipient survival was not significantly influenced by the BMI or any of the AI-derived body composition parameters.

Preoperative assessment of frailty in candidates for kidney transplantation was important as the availability of organs is limited. The BMI is a commonly used indicator of a patient’s fitness although it is an uncertain diagnostic index of obesity [21,22]. If a discrepancy between BMI and obesity is suspected, for example, in patients with sarcopenia and a normal BMI, a more sophisticated assessment is required [23]. Analysis of CT body composition at L3 was shown to allow objective measurement of a patient’s physical fitness [24,25]. In our study, 31% of patients were sarcopenic, which was detected by CT but not BMI. Interestingly, sarcopenia predicted transplant survival in the first year after kidney transplant but not 3-year or 5-year survival. This may have been attributable to the fact that sarcopenia is associated with postoperative complications, which may affect early transplant survival [26]. Three to five years after kidney transplant, the patient’s fitness may have recovered and no longer influenced the outcome [27].

In the past, obesity was considered a risk factor because obese kidney recipients had a higher rate of graft loss and all-cause mortality. Since then, Nicoletto et al. have reported the same graft and patient survival rates for obese and nonobese kidney recipients [28]. Tzvetanov et al. have shown that kidney transplantation is also possible in obese patients [29]. Our results demonstrated that the body composition parameters VAT and SAT, both reflecting the amount of adipose tissue and the cut-off value for obesity, did not aid in risk stratification for overall patient survival. However, VAT emerged as a significant risk factor for 3-year uncensored kidney transplant survival. Consistent with our findings, a recent study of Manabe et al. showed that the visceral fat area is significantly associated with the progression of kidney disease [30]. Moreover, obesity significantly influenced 3-year and 5-year uncensored kidney transplant survival.

The results of our study matched our clinical experience: frailty—represented by the AI-derived body composition parameters SMI/PMI and sarcopenia cut-off value—is an important perioperative risk factor, whereas overweight—represented by the AI-derived body composition parameter VAT and obesity cut-off value—becomes much more relevant for long-term outcomes as it is associated with many cardiovascular diseases. We attributed the differences between censored and uncensored kidney transplant survival to the fact that the latter includes the patient’s death, which may have been influenced by frailty and overweight.

Unlike transplant survival, short- and long-term patient survival was not predicted by any AI-derived body composition parameter or BMI, nor did these parameters aid in risk stratification for overall patient survival. A possible explanation might be that especially older kidney transplant recipients suffered from comorbidities including coronary artery disease, diabetes mellitus, and hypertension, all of which are risk factors for mortality [31,32].

Body composition analysis has been found to be a useful indicator for prognosis and risk stratification, particularly in patients with cardiovascular and malignant diseases [5,33]. Both sarcopenia and sarcopenic obesity are risk factors in cardiovascular disease but also associated with unfavorable outcomes in malignancies and with prolonged postoperative recovery [34,35,36,37]. CT allows for reliable analysis of body composition from a single axial image acquired at the L3 level [15,38]. Most software tools for non-AI-based body composition analysis, such as SliceOmatic or Horos, use pixel thresholding with region growing [39]. However, these manual or semiautomatic segmentation procedures for CT datasets are time consuming and have therefore been limited to smaller patient populations. The postprocessing time of these datasets is dramatically decreased when AI-based body composition analysis is used [40]. In our study, we used an established, fully automatic AI-based software tool that provided valuable metabolic information without additional radiation exposure. In this study, we described the influence of AI-based analysis of body composition parameters on short-term and long-term recipient and transplant survival in older kidney transplant recipients for the first time.

Our study was limited by the use of a retrospective dataset and a relatively small study population. Elderly kidney transplant recipients suffer especially from multiple comorbidities, which may have influenced both graft survival and patient survival as well.

## 5. Conclusions

The survival of kidney recipients cannot be predicted by AI-derived body composition parameters. However, AI-based body composition analysis is useful for predicting short- and long-term kidney transplant survival: the body composition parameters PMI and SMI, both of which represent muscle tissue, and sarcopenia predict 1-year transplant survival, whereas obesity and VAT, which represents fat tissue, predict 3-year and 5-year kidney transplant survival. Therefore, AI-based analysis of body composition may aid in identifying patients at risk who require special care and individualized decision making to achieve optimal outcomes.

## Figures and Tables

**Table 1:** Clinical characteristics of kidney transplant recipients. HLA = human leukocyte antigen. IL-2 = interleukin 2. IS = immunosuppression. KDRI = kidney donor risk index. KDPI = kidney donor profile index. PKD = polycystic kidney disease. * Median ± standard deviation.

	Total (*n* = 42)
Recipient Characteristics	
Recipient age, years *	69 ± 4
Renal disease, *n* (%)	
Diabetic	10 (24%)
Hypertensive	4 (10%)
PKD	5 (12%)
Glomerular disease	9 (21%)
Others/Unknown	14 (33%)
Recipient, female sex, *n* (%)	17 (40%)
Deceased donation, *n* (%)	42 (100%)
Living donation, *n* (%)	0 (0%)
Cold ischemia time, minutes *	581 (241–1076)
Immunosuppression	
Induction IS, *n* (%)	
IL-2 receptor blockade	41 (98%)
Lymphocyte depletion	1 (2%)
Maintenance IS, *n* (%)	
MMF/MPA	42 (100%)
Tacrolimus	41 (98%)
Ciclosporine	1 (2%)
Azathioprin	1 (2%)
Total HLA Mismatches *	3.6 (0–6)
Donor Characteristics	
Donor age, years	69 ± 5
Donor, female sex, *n* (%)	31 (74%)
KDPI (%)	97 ± 3
KDRI	2 ± 0.4

**Table 2:** Patient body composition parameters: BMI = body mass index. PMI = psoas muscle index. SMI = skeletal muscle index. VAT = visceral adipose tissue. SAT = subcutaneous adipose tissue.

Body Composition Parameter	Value (± Standard Deviation)
BMI	27 ± 7
PMI (cm^2^/m^2^)	5.4 ± 1.9
SMI (cm^2^/m^2^)	42.0 ± 7.6
VAT (mm^2^)	203.0 ± 123.3
SAT (mm^2^)	204.7 ± 91.2
Sarcopenia	31%
Obesity	26%
Sarcopenic obesity	12%

**Table 3:** Cox regression analysis of 1-year, 3-year and 5-year patient survival with the variates BMI and AI-derived body composition parameters PMI, SMI, VAT and SAT. AI = artificial intelligence, BMI = body mass index, CI = confidence interval, PMI = psoas muscle index, SMI = skeletal muscle index, SAT = subcutaneous adipose tissue, VAT = visceral adipose tissue.

	1-Year Patient Survival		3-Year Patient Survival		5-Year Patient Survival	
Variate	*p*-Value	Odds Ratio (CI)	*p*-Value	Odds Ratio (CI)	*p*-Value	Odds Ratio (CI)
BMI	0.44	1.03 (0.95–1.12)	0.63	1.03 (0.92–1.14)	0.28	1.05 (0.96–1.15)
PMI	0.64	1.09 (0.76–1.57)	0.49	1.16 (0.76–1.75)	0.40	1.17 (0.81–1.67)
SMI	0.94	1.00 (0.91–1.11)	0.93	0.99 (0.89–1.11)	0.97	1.00 (0.91–1.11)
VAT	1.00	1.00 (0.99–1.01)	0.46	1.00 (0.99–1.01)	0.70	1.00 (0.99–1.01)
SAT	0.91	1.00 (0.99–1.01)	0.63	1.00 (0.99–1.01)	0.99	1.00 (0.99–1.01)

**Table 4:** Cox regression analysis of 1-year, 3-year and 5-year censored kidney transplant survival with the variates BMI and AI-derived body composition parameters PMI, SMI, VAT, and SAT. AI = artificial intelligence, BMI = body mass index, CI = confidence interval, PMI = psoas muscle index, SMI = skeletal muscle index, SAT = subcutaneous adipose tissue, VAT = visceral adipose tissue.

	1-Year Censored Kidney Transplant Survival		3-Year Censored Kidney Transplant Survival		5-Year Censored Kidney Transplant Survival	
Variate	*p*-Value	Odds Ratio (CI)	*p*-Value	Odds Ratio (CI)	*p*-Value	Odds Ratio (CI)
BMI	0.90	1.02 (0.79–1.31)	0.37	1.07 (0.93–1.23)	0.07	1.11 (0.99–1.26)
PMI	0.03	0.25 (0.07–0.89)	0.23	0.62 (0.35–1.29)	0.36	0.79 (0.47–1.31)
SMI	0.03	0.55 (0.35–0.75)	0.05	0.75 (0.50–1.00)	0.06	0.82 (0.65–1.00)
VAT	0.13	0.98 (0.96–1.01)	0.07	1.01 (1.00–1.02)	0.06	0.99 (0.98–1.00)
SAT	0.21	1.01 (0.99–1.03)	0.16	1.00 (1.00–1.01)	0.24	1.01 (1.00–1.01)

**Table 5:** Cox regression analysis of 1-year, 3-year and 5-year uncensored kidney transplant survival with the variates BMI and AI-derived body composition parameters PMI, SMI, VAT and SAT. AI = artificial intelligence, BMI = body mass index, CI = confidence interval, PMI = psoas muscle index, SMI = skeletal muscle index, SAT = subcutaneous adipose tissue, VAT = visceral adipose tissue.

	1-Year Uncensored Kidney Transplant Survival		3-Year Uncensored Kidney Transplant Survival		5-Year Uncensored Kidney Transplant Survival	
Variate	*p*-Value	Odds Ratio (CI)	*p*-Value	Odds Ratio (CI)	*p*-Value	Odds Ratio (CI)
BMI	0.31	1.09 (0.79–1.31)	0.21	1.07 (0.96–1.19)	0.60	1.09 (1.00–1.18)
PMI	0.02	0.26 (0.08–0.84)	0.23	0.76 (0.49–1.19)	0.32	0.83 (0.58–1.20)
SMI	0.01	0.69 (0.46–0.92)	0.06	0.99 (0.98–1.00)	0.08	0.99 (0.99–1.00)
VAT	0.14	0.99 (0.98–1.00)	0.04	1.13 (0.99–1.29)	0.06	0.90 (0.80–1.00)
SAT	0.45	1.00 (0.99–1.02)	0.20	1.00 (1.00–1.01)	0.68	1.00 (0.99–1.01)

**Table 6:** Cox regression analysis of 1-year, 3-year and 5-year patient survival with cutoff values for sarcopenia and obesity. CI = confidence interval.

	1-Year Patient Survival		3-Year Patient Survival		5-Year Patient Survival	
Variate	*p*-Value	Odds Ratio (CI)	*p*-Value	Odds Ratio (CI)	*p*-Value	Odds Ratio (CI)
Sarcopenia	0.10	1.14 (1.01–2.48)	0.76	1.82 (1.24–3.86)	0.68	1.79 (1.26–2.40)
Obesity	0.42	2.19 (0.33–14.7)	0.24	2.12 (0.61–7.39)	0.12	2.39 (0.79–7.23)

**Table 7:** Cox regression analysis of 1-year, 3-year and 5-year censored kidney transplant survival with cutoff values for sarcopenia and obesity. CI = confidence interval.

	1-Year Censored Kidney Transplant Survival		3-Year Censored Kidney Transplant Survival		5-Year Censored Kidney Transplant Survival	
Variate	*p*-Value	Odds Ratio (CI)	*p*-Value	Odds Ratio (CI)	*p*-Value	Odds Ratio (CI)
Sarcopenia	0.10	1.14 (1.01–2.42)	0.10	1.28 (1.06–2.29)	0.31	1.50 (1.13–2.90)
Obesity	0.39	2.31 (0.34–15.6)	0.23	2.53 (0.56–11.3)	0.08	3.31 (0.86–12.7)

**Table 8:** Cox regression analysis of 1-year, 3-year and 5-year uncensored kidney survival with cutoff values for sarcopenia and obesity. CI = confidence interval.

	1-Year Uncensored Kidney Transplant Survival		3-Year Uncensored Kidney Transplant Survival		5-Year Uncensored Kidney Transplant Survival	
Variate	*p*-Value	Odds Ratio (CI)	*p*-Value	Odds Ratio (CI)	*p*-Value	Odds Ratio (CI)
Sarcopenia	0.05	1.19 (1.03–2.02)	0.23	1.51 (1.17–2.53)	0.28	1.60 (1.23–2.52)
Obesity	0.15	3.11 (0.67–14.3)	0.05	3.02 (1.01–9.04)	0.02	2.95 (1.15–7.55)

## Data Availability

The data presented in this study are available on request from the corresponding author.
